# Health and Economic Impact of a Large‐Scale School‐Based Pit and Fissure Sealant Program in Dongguan, China: A Real‐World Program Evaluation

**DOI:** 10.1155/ijod/6899854

**Published:** 2026-08-02

**Authors:** Weiguang Xie, Xuehua Huang, Anzhong Huang, Lin Xu

**Affiliations:** ^1^ Department of Disinfection and Vector Control, Dongguan Center for Disease Control and Prevention, Dongguan, China; ^2^ Department of Applied Health Sciences, University of Birmingham, Birmingham, UK, birmingham.ac.uk; ^3^ School of Public Health, Sun Yat-sen University, Guangzhou, China, sysu.edu.cn; ^4^ School of Public Health, The University of Hong Kong, Hong Kong SAR, China, hku.hk

## Abstract

**Objectives:**

To evaluate the effectiveness and economic impact of a large‐scale school‐based pit and fissure sealant program in Dongguan, China.

**Methods:**

A secondary analysis of deduplicated program data from 9343 students across 20 schools was conducted, comparing sealed and unsealed groups. School‐clustered Poisson and linear probability models were used to estimate associations with caries prevalence and incidence, adjusting for sex, grade, town, and baseline oral conditions. An economic evaluation was performed using project cost data with follow‐up to March 31, 2021. The base‐case analysis adopted a societal perspective with 5% annual discounting.

**Results:**

Compared with unsealed students, the sealed group had lower caries prevalence (adjusted risk ratio [RR] = 0.82; 95% confidence interval [CI]: 0.73–0.92) and lower incident caries (adjusted RR = 0.60; 95% CI: 0.52–0.70). A total of 485,608 teeth were sealed, corresponding to 38,633 prevented carious teeth. The total program cost was 33.99 million yuan, with discounted treatment savings of 115.74 million yuan, yielding a benefit–cost ratio of 3.40. The net cost per prevented carious tooth was −2459.91 yuan, indicating cost savings. In a conservative sensitivity analysis assuming filling‐only treatment, the program was no longer cost‐saving.

**Conclusions:**

The Dongguan school‐based sealant program was associated with reduced caries risk and demonstrated a favorable economic return under base‐case assumptions. These findings support the use of school‐based sealant programs as an effective and potentially cost‐saving strategy for caries prevention in children.

## 1. Introduction

Dental caries is a frequent chronic condition in children and adolescents and remains an important public health problem in China [1, 2]. Despite improvements in oral health over recent decades, the burden of untreated caries in permanent teeth among school‐aged children continues to be substantial, with important implications for pain, infection, and quality of life [3, 4]. First permanent molars are particularly vulnerable shortly after eruption due to their complex occlusal morphology and the difficulty of maintaining adequate oral hygiene at these sites [5].

Pit and fissure sealants are a well‐established and evidence‐based intervention for the prevention of occlusal caries in both primary and permanent teeth [6, 7]. Systematic reviews and randomized trials have consistently demonstrated that sealants can significantly reduce caries incidence, particularly when applied to high‐risk populations and maintained over time [8–10]. In addition, the effectiveness of sealants is influenced by factors such as baseline caries risk, fluoride exposure, and sealant retention, highlighting the importance of program quality and follow‐up in real‐world settings [11].

School‐based sealant programs have been widely promoted as an effective strategy to improve access to preventive dental services, especially for children who may otherwise face barriers to care. Such programs can achieve high coverage, facilitate early intervention at the time of molar eruption, and reduce inequalities in oral health [12]. Evidence from international settings suggests that school‐based sealant programs can be cost‐effective or even cost‐saving, particularly in populations with elevated caries risk and limited access to dental care [13–15]. However, the magnitude of economic benefit is sensitive to assumptions about disease progression and downstream treatment pathways, and real‐world evidence from large‐scale programs in China remains limited [13–15].

In this context, Dongguan implemented a large‐scale school‐based fissure sealant program targeting primary school children, with follow‐up evaluation conducted through March 31, 2021. A comprehensive assessment of both epidemiological effectiveness and economic impact is essential to inform decisions on program continuation, optimization, and scale‐up. Therefore, this study aimed to evaluate the association of sealant application with caries outcomes and assess the cost‐effectiveness of the program using real‐world data from the Dongguan project.

## 2. Methods

### 2.1. Study Design and Data Source

This study was a retrospective secondary analysis of data derived from the Dongguan school‐based pit and fissure sealant program [16]. The analysis used a deduplicated evaluation database constructed from merged program records, together with a project cost workbook documenting resource use and treatment assumptions. The program targeted primary school children and focused on sealing the first permanent molars shortly after eruption. Follow‐up evaluation data were available through March 31, 2021. As the original program was not designed as a randomized trial and most schools included both sealed and unsealed students, the study was treated as an observational comparative evaluation with clustering at the school level.

### 2.2. Ethical Statement

This study was approved by the Institutional Review Board of the Dongguan Center for Disease Control and Prevention (IRB Number 20260001). The analysis was based on the secondary use of deidentified school‐based program evaluation data. No identifiable personal information was available to the investigators for the present analysis. The requirement for individual informed consent was waived by the Institutional Review Board because the study involved a retrospective analysis of deidentified routinely collected program data and posed minimal risk to participants.

### 2.3. Participants

After the removal of duplicate records, a total of 9343 students from 20 schools were included in the analytic dataset. Of these, 7090 students received pit and fissure sealants and were classified as the sealed group, while 2253 students did not receive sealants and served as the comparison group. Students were included if they had complete baseline and follow‐up examination records for first permanent molars. The comparison between groups reflected routine program implementation rather than random allocation, thereby representing real‐world conditions.

### 2.4. Outcomes

The primary epidemiological outcomes were caries prevalence and incident caries in the first permanent molars. Caries prevalence was defined as the presence of carious lesions at follow‐up, while incident caries referred to newly developed carious lesions during the follow‐up period among teeth at risk at baseline. Baseline oral health status was characterized using the number of carious surfaces and the number of deep‐pit surfaces, both of which were included as covariates in the analysis to account for underlying risk differences.

### 2.5. Statistical Analysis

Associations between sealant application and caries outcomes were assessed using regression models that accounted for clustering at the school level. Poisson regression models with robust variance estimation were used to estimate adjusted risk ratios (RRs), and linear probability models were used to estimate absolute risk differences. All models were adjusted for sex, grade, town, baseline carious surface count, and baseline deep‐pit surface count. Statistical analyses were conducted using R software (Version 4.5.2; R Foundation for Statistical Computing, Vienna, Austria), and a two‐sided *p*‐value of less than 0.05 was considered statistically significant.

### 2.6. Economic Evaluation

An economic evaluation was conducted using data from the project cost workbook, incorporating program costs, treatment pathways, and estimates of prevented carious teeth. The program sealed 218,434 teeth in 2017 and 267,174 teeth in 2018. The workbook recorded 14,854 and 23,779 prevented carious teeth for the respective cohorts, and these values were used as the base‐case effectiveness measure because they were directly linked to the program’s benefit calculations. Costs and outcomes were discounted to March 31, 2021, using an annual discount rate of 5%, in accordance with Chinese pharmacoeconomic guidelines [17]. The primary analysis adopted a societal perspective, including both direct treatment costs and caregiver productivity loss, while a secondary analysis from a healthcare‐system perspective excluded productivity loss. A conservative sensitivity analysis was performed assuming that each prevented carious tooth would have required only a dental filling. In addition, one internally inconsistent cost entry in the 2018 workbook was recalculated based on the stated unit costs prior to the final analysis.

## 3. Results

A total of 9343 students from 20 schools were included in the deduplicated dataset, of which 19 schools contained both sealed and unsealed students. The baseline characteristics of the analytic sample are presented in Table 1. At follow‐up, caries prevalence was lower in the sealed group than in the unsealed group (28.7% vs. 41.2%), and the proportion of incident caries during follow‐up was also lower (17.2% vs. 30.2%). Detailed comparative results are presented in Table 2.

**Table 1:** Baseline characteristics of students included in the deduplicated analytic dataset, overall and by sealant status.

Characteristic	Overall (*N* = 9343)	Sealed (*n* = 7090)	Unsealed (*n* = 2253)	Absolute SMD^a^
Males, *n* (%)	5,166 (55.3)	3912 (55.2)	1254 (55.7)	0.01
Grade 6 at follow‐up, *n* (%)	4,498 (48.1)	3176 (44.8)	1322 (58.7)	0.28
Any baseline caries, *n* (%)	1,595 (17.1)	1062 (15.0)	533 (23.7)	0.22
Baseline carious surface count, mean (SD)	0.40 (1.19)	0.30 (0.96)	0.71 (1.69)	0.30
Any baseline deep‐pit surface, *n* (%)	8,480 (90.8)	7,050 (99.4)	1430 (63.5)	1.04
Baseline deep‐pit surface count, mean (SD)	6.01 (6.26)	6.68 (6.35)	3.92 (5.45)	0.47

^a^SMD indicates standardized mean difference; absolute values greater than 0.10 generally suggest meaningful between‐group imbalance.

**Table 2:** Cluster‐adjusted comparative effectiveness of the Dongguan school‐based fissure sealant program, follow‐up through March 31, 2021.

Outcome	Sealed group (%)	Unsealed group (%)	Adjusted RR^a^ (95% CI)	*p*‐Value for RR	Adjusted RD^a^ (95% CI), percentage points	*p*‐Value for RD
Caries prevalence	28.7	41.2	0.82 (0.73–0.92)	<0.001	−6.1 (−9.8 to −2.3)	0.002
Incident caries	17.2	30.2	0.60 (0.52–0.70)	<0.001	−11.5 (−14.4 to −8.7)	<0.001

Abbreviations: CI, confidence interval; RD, risk difference; RR, risk ratio.

^a^Adjusted for sex, grade, town, baseline carious surface count, and baseline deep‐pit surface count; confidence intervals and *p*‐values were estimated with school‐clustered robust variance.

After adjustment for school clustering and baseline differences, sealant application was associated with a reduced risk of caries prevalence (adjusted RR = 0.82; 95% confidence interval [CI]: 0.73–0.92) and incident caries (adjusted RR = 0.60; 95% CI: 0.52–0.70). The corresponding absolute risk differences were −6.1 percentage points for caries prevalence and −11.5 percentage points for incident caries (Table 2).

In the economic evaluation, the combined 2017 and 2018 cohorts incurred a total program cost of 33.99 million yuan and generated discounted treatment savings of 115.74 million yuan under the societal perspective, resulting in a net benefit of 81.74 million yuan and a benefit–cost ratio of 3.40. The gross cost per prevented carious tooth was 1022.94 yuan, while the net cost per prevented carious tooth was −2459.91 yuan.

From the healthcare‐system perspective, discounted treatment savings were 96.37 million yuan, yielding a net benefit of 62.38 million yuan and a benefit–cost ratio of 2.84. In the conservative sensitivity analysis assuming filling‐only treatment, discounted treatment savings decreased to 16.04 million yuan, and the benefit–cost ratio declined to 0.47, with a net cost of 540.32 yuan per prevented carious tooth. Base‐case findings under different analytic scenarios are summarized in Table 3.

**Table 3:** Cost‐effectiveness results for the Dongguan school‐based fissure sealant program: base‐case and sensitivity analyses, follow‐up through March 31, 2021.

Scenario	Program cost (yuan)	Discounted treatment savings (yuan)	Net benefit (yuan)	BCR	Discounted prevented carious teeth	Gross cost per prevented tooth (yuan)	Net cost per prevented tooth (yuan)^b^
Base case^a^	33,992,560.00	115,735,863.00	81,743,303.00	3.40	33,230.14	1,022.94	−2,459.91
Healthcare‐system perspective	33,992,560.00	96,369,057.06	62,376,497.06	2.84	33,230.14	1,022.94	−1,877.11
Conservative filling‐only sensitivity	33,992,560.00	16,037,528.43	−17,955,031.57	0.47	33,230.14	1,022.94	540.32
Incidence‐based effect‐count sensitivity	33,992,560.00	184,702,211.86	150,709,651.86	5.43	53,031.79	640.98	−2,841.87

Abbreviation: BCR, benefit–cost ratio.

^a^Base case used the societal perspective, workbook‐defined prevented carious teeth counts, and 5% annual discounting through March 31, 2021.

^b^Negative net cost per prevented tooth indicates cost saving.

## 4. Discussion

In this large school‐based program evaluation, pit and fissure sealant application was associated with lower caries prevalence and reduced incidence of new carious lesions in first permanent molars under real‐world conditions. In addition to these epidemiological benefits, the program demonstrated a favorable economic profile in the base‐case analysis, with substantial treatment cost savings and a benefit–cost ratio greater than one. However, the magnitude of economic benefit was sensitive to assumptions regarding downstream treatment pathways, as reflected by the conservative sensitivity analysis.

The epidemiological findings of this study are consistent with a substantial body of evidence demonstrating that pit and fissure sealants are effective in reducing occlusal caries in permanent molars [6, 7, 18, 19]. Previous clinical trials and systematic reviews have shown that sealants can significantly lower caries risk, particularly when applied to newly erupted molars and maintained over time [7, 9, 20, 21]. The observed associations in this study, derived from a large‐scale real‐world program, further support the effectiveness of sealants under routine implementation conditions. In addition, our findings are in line with prior municipal evidence from Guangzhou, which reported sustained reductions in caries following school‐based sealant delivery [22], suggesting that such programs can achieve consistent benefits across different urban settings in China. The economic results are also broadly consistent with international evidence indicating that school‐based sealant programs can be cost‐effective or cost‐saving, particularly in populations with elevated caries risk and limited access to dental care [12, 23, 24]. Previous economic evaluations have highlighted that the cost‐effectiveness of sealant programs depends on factors such as baseline disease risk, program coverage, and assumptions regarding avoided treatment pathways [24, 25]. The present study adds to this literature by providing empirical evidence from a large‐scale program in China, demonstrating that substantial economic benefits can be achieved under plausible real‐world assumptions.

These findings have several implications for population‐level caries prevention. The observed reductions in both caries prevalence and incident disease support the effectiveness of school‐based sealant delivery when implemented at scale under routine conditions. In this study, the base‐case cost savings were driven by the linkage of prevented lesions to higher‐cost pulpitis treatment, whereas a more conservative filling‐only assumption substantially reduced the estimated economic benefit. This pattern highlights that economic evaluations of preventive interventions are inherently sensitive to clinical management pathways and underlying risk distributions, rather than indicating uncertainty about the preventive effect itself.

Several limitations should be considered when interpreting these findings. First, the analysis was based on observational program data rather than a randomized design. Although the regression models adjusted for sex, grade, town, baseline carious surface count, and baseline deep‐pit surface count, allocation to sealant receipt occurred through routine program implementation rather than random assignment. Therefore, unmeasured differences between sealed and unsealed students, including oral hygiene practices, parental preferences, access to dental care, school‐level implementation factors, and clinician decision‐making, may have contributed to the observed differences in caries outcomes. Second, because most schools included both sealed and unsealed students, the comparison does not represent a true cluster‐randomized evaluation and may have attenuated school‐level differences. Third, incomplete recording of intervention dates required imputation, which could have introduced minor uncertainty into discounted cost and outcome estimates. Fourth, the economic findings are more assumption‐dependent than the epidemiological associations. In the base‐case analysis, the estimated savings were driven by the workbook assumption that a proportion of prevented lesions would otherwise progress to more costly treatment pathways. Finally, the economic results relied on workbook‐based assumptions regarding avoided treatment pathways; if the proportion of lesions progressing to more severe disease is lower in routine practice, the estimated cost savings would be correspondingly reduced. Therefore, these results should not be interpreted as showing that all school‐based sealant programs will be cost‐saving in all settings. Economic efficiency is likely to vary according to baseline caries risk, disease severity, sealant retention, program delivery costs, local treatment prices, and usual clinical management patterns.

From a policy perspective, these findings support the continued implementation and optimization of school‐based sealant programs as part of comprehensive oral health strategies for children. However, the observational design and assumption‐dependent economic estimates indicate that decisions about scale‐up should be accompanied by strengthened monitoring systems. Future program data should capture sealant retention, lesion severity, referral completion, treatment actually received, and longer‐term follow‐up, which would allow more robust causal and economic evaluation.

## 5. Conclusion

School‐based pit and fissure sealants were associated with substantially lower caries prevalence and incidence in first permanent molars under real‐world conditions. The program was cost‐saving under base‐case assumptions but sensitive to treatment pathway assumptions. These findings support school‐based sealant delivery as an effective and potentially efficient strategy for caries prevention in low‐ and middle‐income settings with a high unmet need.

## Author Contributions

**Weiguang Xie**: conceptualization, data curation, formal analysis, investigation, methodology, project administration, writing – original draft. **Xuehua Huang**: data curation, investigation, project administration, resources, validation, writing – review and editing. **Anzhong Huang**: investigation, resources, validation, writing – review and editing. **Lin Xu**: conceptualization, methodology, supervision, formal analysis, writing – original draft, writing – review and editing.

## Funding

This study did not receive any specific funding.

## Disclosure

All authors have read, reviewed, and approved the final version of the manuscript. Lin Xu, as the corresponding author and manuscript guarantor, had full access to all of the data in this study and takes complete responsibility for the integrity of the data and the accuracy of the data analysis.

## Ethics Statement

This study was approved by the Institutional Review Board of the Dongguan Center for Disease Control and Prevention (IRB Number 20260001). The analysis was based on the secondary use of deidentified program evaluation data, and the requirement for informed consent was waived.

## Conflicts of Interest

The authors declare no conflicts of interest.

## Data Availability

The authors confirm that the aggregated data supporting the findings of this study are available within the article and/or its Supporting Information. Individual‐level program data cannot be publicly shared because they were derived from school‐based health program records and are subject to institutional data governance restrictions. Reasonable requests for access to deidentified analytic data may be directed to the corresponding author and will be considered subject to approval by the Dongguan Center for Disease Control and Prevention.
